# Some virulence genes are associated with antibiotic susceptibility in *Enterobacter cloacae* complex

**DOI:** 10.1186/s12879-024-09608-2

**Published:** 2024-07-19

**Authors:** Fatemeh Mosaffa, Fereshteh Saffari, Mahin Veisi, Omid Tadjrobehkar

**Affiliations:** Departement of Medical Microbiology (Bacteriology & Virology), Afzalipour School of Medicine, Kerman University of Medical Sciences, Kerman, Iran

**Keywords:** *Enterobacter cloacae* complex, Virulence gene, Antibiotic resistance, MDR, Antivirulence

## Abstract

**Background:**

*Enterobacter cloacae* complex (ECC) including different species are isolated from different human clinical samples. ECC is armed by many different virulence genes (VGs) and they were also classified among ESKAPE group by WHO recently. The present study was designed to find probable association between VGs and antibiotic susceptibility in different ECC species.

**Methods:**

Forty-five *Enterobacter* isolates that were harvested from different clinical samples were classified in four different species. Seven VGs were screened by PCR technique and antibiotic susceptibility assessment was performed by disk-diffusion assay.

**Result:**

Four *Enterobacter* species; *Enterobacter cloacae* (33.3%), *Enterobacter hormaechei* (55.6%), *Enterobacter kobei* (6.7%) and *Enterobacter roggenkampii* (4.4%) were detected. Minimum antibiotic resistance was against carbapenem agents and amikacin even in MDR isolates. 33.3% and 13.3% of isolates were MDR and XDR respectively. The *rpoS* (97.8%) and *csgD* (11.1%) showed maximum and minimum frequency respectively. Blood sample isolated were highly virulent but less resistant in comparison to the other sample isolates. The *csgA*, *csgD* and *iutA* genes were associated with cefepime sensitivity.

**Conclusion:**

The *fepA* showed a predictory role for differentiating of *E. hormaechei* from other species. More evolved iron acquisition system in *E. hormaechei* was hypothesized. The *fepA* gene introduced as a suitable target for designing novel anti-virulence/antibiotic agents against *E. hormaechei*. Complementary studies on other VGs and ARGs and with bigger study population is recommended.

## Introduction

*Enterobacter* genus is a facultative anaerobic, Gram-negative rod-shape bacterium which categorized in the family *Enterobacteriaceae*. The taxonomy of *Enterobacter* is complicated and modified continuously, many members of *Enterobacter* genus were moved to the other genus recently [1, 2]. The species that collectively classified as *Enterobacter cloacae complex (ECC)* are the most reported pathogens form human infections [3, 4]. Recently, whole-genome sequencing investigations suggested possible reclassification of *Enterobacter aerogenes* as *Klebsiella aerogenes* or *Klebsiella mobilis*. However, there are many morphological and biochemical differences between two genera [1, 5]. Several species were reported from ECC till now. *Enterobacter cloacae*, *Enterobacter asburiae*, *Enterobacter hormaechei*, *Enterobacter kobei*, *Enterobacter ludwigii*, and *Enterobacter nimipressuralis* were introduced as most common isolates from human clinical samples [6].

But, taxonomy of *Enterobacter* is updating continuously and precise identification of *Enterobacter* species has a multistep challenging, expensive and time-consuming protocol. The ECC members identified primarily through Matrix-assisted laser desorption/ionization -Time of flight (MALDI-TOF) mass spectrometry (MS) technique and it usually will be completed by some molecular methods [3, 7]. Recently Yang et al., introduced a one-step multiplex PCR method for differential identification of four member of ECC, including; *E cloacae*, *E hormaechei*, *E kobei* and *E roggenkampii* [7].

Numerous virulence genes were detected in *Enterobacter* species including gene encoding different adhesins, biofilm related genes, iron acquisition system genes, stress response genes, and various secretion system attributed genes [8–10].

Type I fimbriae was detected in different Gram-negative uropathogens. This adhesin is responsible for binding of bacterium to mannosylated receptor molecules on the uroepithelium. It is encoded by *fim*ACDFIHZ locus and FimH is the fimbriae tip located molecule [11]. Another major adhesin of *Enterobacter* is curli fimbriae. The major construction subunit of curli is CsgA that encoded by *csgBA*C operon and another homologous operon *csgDEFG* encode transcription activator CsgD and two incorporated chaperons. Curli fimbriae were reported from other genus of *Enterobacteriaceae*, it is involved in adhesion and biofilm formation [12–14]. Several Enterobacter species were well known for their inhibitory actions against plant pathogens, that is attributed to the competition on iron uptake from soil due to production of different siderophores [15]. Enterobactin, aerobactin and yersiniabactin were reported from Enterobacter [16]. Ferric enterobactin receptor (FepA) is a multifunctional outer membrane protein that is detected in different genera of *Enterobacteriaceae*. It is the receptor for colicins, enterobactin iron complex and also it could be used by some bacteriophages for entry to bacterial cell [17, 18]*.* The *iutA* is encoding gene of ferric aerobactin receptor [19]. Flagellum specific ATP-synthetase is encoded by *fliI* gene that plays a critical role in stress adaptation and pathogenesis of *E. cloacae* [20]. Another gene that plays role in stress responses of *Enterobacter* species is *rpoS* gene. It is RNA polymerase sigma factor encoding gene that has some different homologues genes in different bacteria [21].

In 2017 *Enterobacter* species were placed in ESKAPE group (*Enterococcus faecium, Staphylococcus aureus, Klebsiella pneumoniae, Acinetobacter baumannii, Pseudomonas aeruginosa, and Enterobacter species*) by WHO, that they are the most critical antibiotic resistance bacteria and were represented as very high level threats for human kind [4]. Different types of extended spectrum β-lactamases (ESBLs) and carbapenemases were detected from *Enterobacter* species that cause intrinsic resistance to wide range of cell wall synthesis active antibiotics. Many strains of *Enterobacter* are regarded as multidrug-resistant (MDR). The colistin was used as the one of the few effective antibiotic agents against these MDR strains, but recently colistin resistant isolates were reported repeatedly worldwide [22–24]. Therefore, continuous research in order to finding new alternatives is a constant need. Some of the studies reported that anti-virulence agents could be suitable candidates in combating against antibiotic resistant infections [25–28]. In the present study we tried to investigate antibiotic susceptibility profile and also a list of virulence genes of *Enterobacter* species. We also tried to discover the possible logical relationships between antibiotic resistance and virulence-related genes in order to introduce new targets for planning future anti-virulence strategies.

## Material and methods

### Specimen collection and *bacteria* isolation

In this study, a total of 53 laboratory isolates with initial diagnosis of *Enterobacter* were collected from different samples of hospitalized patients referred to three medical centers affiliated to Kerman University of Medical Sciences in Kerman. All bacterial isolates were cultivated during routine diagnosis and treatment protocols, not by means of research. We only collect the grown media unknown. Finally, 45 out of 53 isolates were confirmed as a distinct *Enterobacter* species in subsequent steps and 8 unknown species were omitted from the study.

The isolates were obtained from different samples (urine, burning wound, surgical wound, upper respiratory tract and blood). Confirmatory identification and also species determination took placed in the bacteriology laboratory of Afzalipour school of medicine.

### Species identification

The bacterial isolates were primarily identified by standard biochemical tests. Swarming on blood agar, monosaccharide fermentation pattern, H_2_S production in triple sugar iron agar, Indole test, Methyl Red-Voges Proskauer test, citrate utilization test, urease test, lysine decarboxylase test and ornithine decarboxylase test were used in identification of genus and also species differentiation. Species identification take placed through PCR amplification by species-specific primers [7]. Finally, four *Enterobacter* species(*E cloacae*, *E hormaechei*, *E kobei* and *E roggenkampii*) were tracked by specific primers through PCR technique. The confirmed isolates were inoculated into the tryptic soy broth enriched with 20% glycerol and stored at -70 °C for next steps experiments.

### Antimicrobial susceptibility assessment

Disk diffusion method was used to determine antibiotic susceptibility of the isolates according to the guidelines of the Institute of Clinical and Laboratory Standards (CLSI) and supplementary reviews [29, 30].

Antibiotic discs were provided from Padtan-Teb.Co (Iran) and were as following: Amoxicillin (25 µg), Ceftazidime (30 µg), Ceftriaxone (30 µg), Cefotaxime (30 µg), Aztreonam (30 µg), Imipenem (10 µg), Meropenem (10 µg), Tobramycin (10 µg), Norfloxacin (10 µg), Gentamicin (10 µg), Cefalexin (30 µg), Trimethoprim/Sulfamethoxazole (1.23 /25.75 µg), Cefoxitin(30 µg), Amikacin (30 µg), Cefepime (30 µg), Cefuroxime (30 µg), Tetracycline (30 µg).

Multidrug-resistance (MDR) and extremely drug resistant (XDR) isolates were detected by CLSI guideline [30].

### Bacterial genome lysate preparation

Lysate preparation was performed by boiling method. In summary, a loopful of an overnight culture of bacterial isolates were inoculated into the microtubes including of 500 µl of distilled water. The microtubes were places in water bath at 100 °C for 10 min. In the next step, the sample was centrifuged (12,000 rpm for 5 min). After centrifugation, the supernatant was separated and kept in -70 °C.

### Polymerase chain reaction conditions and primers list for detection of virulence genes (VGs)

The presence of some virulence genes was investigated by polymerized by chain reaction method through Biometra thermocycler (Germany) and specific primers (Table 1).

**Table 1:** List of the primers that used for screening of VGs through PCR technique

Gene	Primer sequence (5′ to 3′)	Product size (bp)	Gene Description
*csgA*	F CTGACGACAGCACCATCTCT R TCCACCGTACTGGCTCACAT	107	Curli fimbriae major subunit (CsgA)
*csgD*	F AGGCCTTCTACCACCCGATTC R GACGAGTATCCTTTCCGGGAC	92	CsgBAC operon transcriptional regulatory protein
*fliI*	F ACTGCCATCTGTTCGTCGTT R AGTCTTTAACTTCGCGGCCA	593	The flagellum-specific ATP-synthase
*rpoS*	F AATCTCTTCTGCGCTTGGCT R TGCTTTGCGTGGTGATGTTG	349	RNA polymerase sigma factor (RpoS)
*fimH*	F AGGAACAACCGGAAAGTCCA R TTCGCCACGACAAACCCTAA	621	Encodes a protein at the tip of fimbriae type I
*fepA*	F TCTTTT TTCACCGGCATGGA R CGTGCGGTGGTCAATATCT	572	Ferric enterobactin receptor
*iutA*	F TGAAACGTTCTCATCTTTGGGT R TCG AAGGTTTCATGGTCGGC	1098	Ferric aerobactin receptor

PCR products were separated by gel electrophoresis using 1.5% agarose gel with 100 bp DNA Ladder.

### Statistical analysis

Data analysis was performed using SPSS 19 statistical software. Chi-square and Fisher’s exact test were used to analysis differences in antibiotic resistance between four studied *Enterobacter* species and also the association between antibiotic resistance against different antibiotic agents with other studied variables such as; virulence genes, multidrug-resistance. Binary logistic regression analysis was used to evaluate the predictory role of virulence genes for resistance to different antibiotic agents. The *p* ≤ 0.05 was considered as significant.

## Results

### Distribution of bacterial isolates in different samples

Totally 45 *Enterobacter* species were identified in this study. Fifteen (33.3%) *Enterobacter cloacae*, 25 (55.6%) *Enterobacter hormaechei*, 3 (6.7%) *Enterobacter kobei* and 2 (4.4%) *Enterobacter roggenkampii* were confirmed. These known isolates were used in the all experiments. Thirteen isolates were obtained from surgical wound (28.9%), 10 isolates (22.2%) were harvested from upper respiratory tract (bronchoalveolar lavage fluid and throat swabs). Eleven (24.4%), 7 (15.6%) and 4 (8.9%) of isolates were harvested from blood culture, urine and burning wounds respectively. Frequency of different species was not significantly different among various samples (Pearson Chi-square, *p* = 0.189). Data are presented in Table 2.

**Table 2:** Frequency of identified Enterobacter species obtained from different samples

Species	Surgical wound *n*(%)	Blood culture *n*(%)	URT *n*(%)	Urine culture *n*(%)	Burning wound *n*(%)
***E. hormaechei***	5(38.5))	6(54.5)	8(80)	4(57.1)	2(50)
***E. cloacae***	3(23.1)	5(45.5)	2(20)	3(42.9)	2(50)
***E. kobei***	3(23.1)	0(0)	0(0)	0(0)	0(0)
***E. roggenkampii***	2(15.4)	0(0)	0(0)	0(0)	0(0)
**Total**	13(100)	11(100)	10(100)	7(100)	4(100)

### Frequency of antibiotic resistance among the identified *Enterobacter* species

**Fig. 1:**
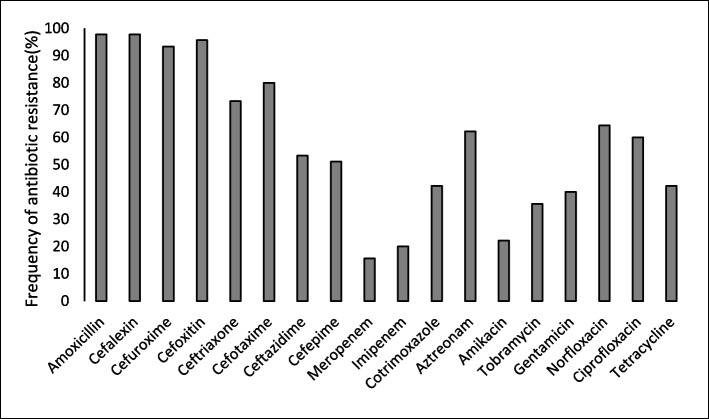
Frequency of resistance against different antibiotic agents in Enterobacter isolates. The trimethoprim/sulfamethoxazole combination referred as Cotrimoxazole

Data collected from disk diffusion test showed different frequency of resistance against various antibiotic agents (Fig. 1) and also antibiotic resistance was different among four studied *Enterobacter* species (Table 3). But, by means of reach a trustable finding the frequency of antibiotic resistant isolates for each species was compared with same frequency in collection of other species separately through Pearson Chi-square analysis. Results showed that, ceftriaxone resistance was significantly more prevalent among *E. cloacae* isolates (93.8% vs 70.3%, *p* = 0.049) and also less prevalent among *E. hormaechei* isolates (60% vs 92.9%, *p* = 0.004) in comparison to the other isolates. Significant less frequency of cefotaxime resistance (68% vs 96.4%, *p* = 0.007), aztreonam resistance (52% vs 78.6%, *p* = 0.041) and tobramycin resistance (24% vs 53.6%, *p* = 0.028) also were detected in *E. hormaechei* isolates in comparison to the other isolates. Binary logistic regression analysis also proposed a positive predictory expression of ceftriaxone resistance in *E. cloacae* isolates ( *p* = 0.016. odds ration = 2.278). Resistance against other antibiotic agents was not significantly different in four studied *Enterobacter* species.

**Table 3:** Frequency of resistance against different antibiotic agents in four Enterobacter species

Antibiotics	Frequency of antibiotic resistant isolates *n*(%)			
	*E. cloacae*	*E. hormaechei*	*E. kobei*	*E. roggenkampii*
Amoxicillin	15(100)	24(96)	3(100)	2(100)
Cephalexin	15(100)	24(96)	3(100)	2(100)
Cefuroxime	15(100)	22(88)	3(100)	2(100)
Cefoxitin	14(93.3)	24(96)	3(100)	2(100)
Ceftriaxone	14(93.3)	15(60)	2(66.7)	2(100)
Cefotaxime	14(93.3)	17(68)	3(100)	2(100)
Ceftazidime	10(66.7)	11(44)	2(66.7)	1(50)
Cefepime	9(60)	11(44)	2(66.7)	1(50)
Imipenem	9(60)	9(36)	1(33.3)	2(100)
Meropenem	3(20)	3(12)	1(33.3)	0(0)
Cotrimoxazole	5(33.3)	12(48)	1(33.3)	1(50)
Aztreonam	11(73.3)	13(52)	2(66.7)	2(100)
Amikacin	4(26.7)	4(16)	2(66.7)	0(0)
Tobramycin	7(46.7)	6(37.5)	2(66.7)	1(50)
Gentamicin	7(46.7)	8(32)	2(66.7)	1(50)
Norfloxacin	11(73.3)	16(64)	1(33.3)	1(50)
Ciprofloxacin	8(53.3)	16(64)	1(33.3)	2(100)
Tetracycline	7(46.7)	11(44)	1(33.3)	0(0)

The trimethoprim/sulfamethoxazole combination referred as Cotrimoxazole

Cumulative resistance against ceftriaxone, gentamicin and ciprofloxacin were regarded as multidrug-resistant and XDR isolates had extra resistant to meropenem in comparison to MDR isolates. Fifteen (33.3%) isolates were MDR and XDR was detected in 6 (13.3%) isolates. Pearson chi-square analysis (*p* ≤ 0.05) showed higher resistance against cefotaxime, ceftazidime, cefepime, trimethoprim/sulfamethoxazole (cotrimoxazole), imipenem, meropenem, tobramycin, amikacin, norfloxacin, aztreonam and tetracycline in MDR isolates in comparison to the non-MDRs (Table 4).

**Table 4:** Frequency of antibiotic resistance among MDR isolates in comparison to the non-MDR isolates

**Antibiotics**	**Non-MDR** *n*(%)	**MDR** *n*(%)	***p-value***
Amoxicillin	29(96.7)	15(100)	0.475
Cephalexin	19(96.7)	15(100)	0.475
Cefuroxime	27(90.0)	15(100)	0.205
Cefoxitin	28(93.3)	15(100)	0.306
Ceftriaxone	18(60.0)	15(100)	0.004
Cefotaxime	21(70.0)	15(100)	**0.018**
Ceftazidime	9(37.5)	15(62.5)	**0.000**
Cefepime	8(26.7)	15(100)	**0.000**
Imipenem	3(10.0)	6(40.0)	**0.018**
Meropenem	1(3.3)	6(40.0)	**0.001**
Cotrimoxazole	8(26.7)	11(73.3)	**0.003**
Aztreonam	13(43.3)	15(100)	**0.000**
Amikacin	3(10.0)	7(46.7)	**0.005**
Tobramycin	3(10.0)	13(86.7)	**0.000**
Gentamicin	3(10.0)	15(100)	0.000
Norfloxacin	16(53.3)	13(86.7)	**0.028**
Ciprofloxacin	12(40.0)	15(100)	0.000
Tetracycline	7(23.3)	12(80.0)	**0.000**

Cumulative resistance against ceftriaxone, gentamicin and ciprofloxacin regarded as MDR. The trimethoprim/sulfamethoxazole combination referred as Cotrimoxazole. The analysis was performed by Pearson Chi-square test and *p* ≤ 0.05 was regarded as significant

Pearson chi-square analysis also showed, significant higher frequency of resistance against ceftazidime (100% vs 46.2%, *p* = 0.014), cefepime (100% vs 43.6%, *p* = 0.010), imipenem (83.3% vs 10.3%, *p* = 0.000), aztreonam (100% vs 56.4%, *p* = 0.040) amikacin (100% vs 10.3%, *p* = 0.000) and tobramycin (100% vs 25.6%, *p* = 0.000) among XDR isolates in comparison to the other isolates.

Pearson chi-square test analysis showed that, frequency of resistance against ciprofloxacin was significantly different (*p* ≤ 0.05) in the isolates which were recovered from different sources, URT samples (33.3%), urine (18.5%) and surgical wound (29.6%), blood (18.5%) and burning wound (0%). There were not significant differences in the frequency of resistance against other antibiotics among samples taken from different wards and different sources.

### Frequency of different VGs in the studied isolates

All of the virulence genes were detected in studied isolates. The *rpoS* and *csgD* were most frequent and less common genes respectively. The *rpoS* was detected in 44 (97.8%) out of 45 isolates and *csgD* was detected only in 5 (11.1%) isolates. The frequency of other virulence genes were as follow; *csgA* 7 (15.6%), *fliI* 40 (88.9%), *fimH* 21 (46.7%), *fepA* 35 (77.8%) and *iutA* 40 (88.9%).

Pearson Chi-square analysis revealed that prevalence of *iutA* and *fepA* genes was significantly different among four *Enterobacter* species and they were more frequent in *E. hormaechei* isolates in comparison to the other isolates (Table 5). Binary logistic regression analysis also proposed a positive predictory role of *fepA* gene ( *p* = 0.020. odds ration = 2.216) for differentiation of *E. hormaechei* from other species and also a negative predictory role of *csgD* gene ( *p* = 0.026. odds ration = -2.686) for differentiation of *E. hormaechei* from other species. Three (6.7%) isolates (two *E. cloacae* and one *E. hormaechei*)were armed by all studied virulence genes. Statistical analysis showed that all of these highly virulent isolates were non-MDR and they were susceptible to majority of studied antibiotic agents including; cefepime, imipenem, meropenem, tobramycin, gentamicin, amikacin, trimethoprim/sulfamethoxazole and tetracycline.

**Table 5:** Frequency of virulence genes among different Enterobacter species

Virulence genes	*Enterobacter* species *n*(%)				*p-value*
	***E. cloacae*** **15(100)**	***E. hormaechei*** **25(100)**	***E. kobei*** **3(100)**	***E. roggenkampii*** **2(100)**	
***csgA***	3(20)	3(12)	0(0)	1(50)	0.419
***csgD***	3(20)	1(4)	0(0)	1(50)	0.116
***fliI***	15(100)	22(88)	2(66.7)	1(50)	0.091
***fimH***	7(46.7)	12(48)	1(33.3)	1(50)	0.971
***rpoS***	15(100)	24(96)	3(100)	2(100)	0.845
***fepA***	**9(60)**	**23(92)**	**2(66.7)**	**1(50)**	**0.039**
***iutA***	**12(80)**	**25(100)**	**2(66.7)**	**1(50)**	**0.031**

The comparison was performed by Pearson Chi-square analysis and *p* ≤ 0.05 was regarded as significant

### Frequency of different virulence genes regarding to the antibiotic resistance in the studied isolates

**Fig. 2:**
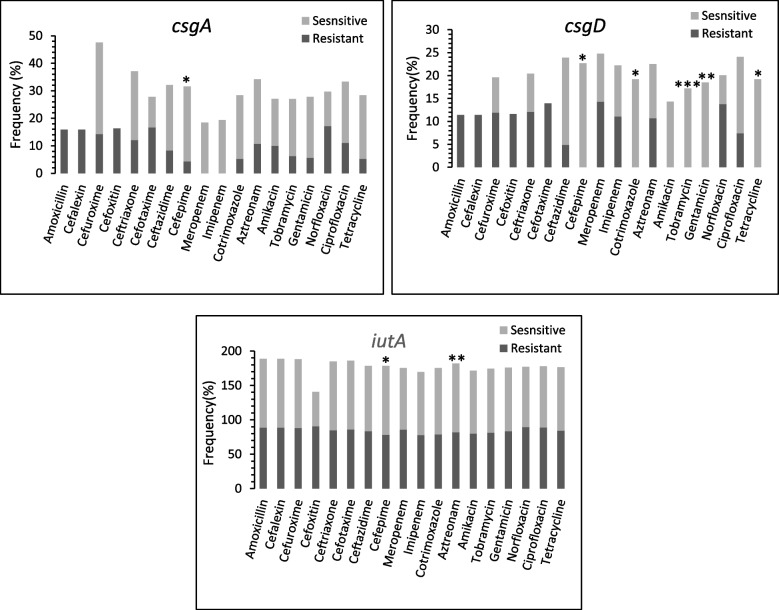
Frequency of csgA, csgD and iutA genes among antibiotic resistance isolates in comparison to the antibiotic sensitive isolates. The trimethoprim/sulfamethoxazole combination referred as Cotrimoxazole. The *p*-value was derived from Fischer’s exact test analysis. *p ≤ 0.05, **p ≤ 0.06 and ***p ≤ 0.09

Results obtained from Chi-square analysis showed different frequency of *csgA*, *csgD*, and *iutA* genes among antibiotic resistant isolates in comparison to antibiotic sensitive isolates (Fig. 2). While, the frequency of the other studied virulence genes was not significantly different between these two groups (data not shown).

The Fischer’s exact test analysis showed that *csgA* gene was more prevalent among cefepime sensitive isolates in comparison to the cefepime resistant isolates (27.3% vs 4.3%, *p* = 0.042). Similar finding was also observed for *csgD* (22.7% vs 0%, *p* = 0.022) and *iutA* (100% vs 78.3%, *p* = 0.028) genes. The *csgD* gene also had higher frequency among the isolates that were sensitive against trimethoprim/sulfamethoxazole combination (19.2% vs 0%, *p* = 0.043) and tetracycline (19.2% vs 0%, *p* = 0.043) in comparison to the resistant isolates (Fig. 2).

## Discussion

Resistance against third generation cephalosporins and carbapenem antibiotics among *Enterobacter* species were recently categorized as priority 1 or critical by WHO experts, which had to considered in prescription of antibacterial agents [31]. Therefore, continuous screening of antibiotic resistance pattern and also finding new targets for development of new drugs with antibacterial or anti-virulence properties is mandatory.

Maximum resistance was detected against penicillin antibiotics and also first and second generation cephalosporins in the present study. High level of resistance against third generation cephalosporins also was observed (Fig. 1). Expression of chromosomal *ampC* gene as intrinsic mechanism could be responsible of resistance against penicillin antibiotics and also first and second generation cephalosporins in the studied isolates. Continuous hyperproduction of AmpC and also plasmid encoded ESBLs were probable major factors behind resistance against third generation cephalosporins and aztreonam as it reported recently [32].

Data obtained from the present study showed that minimum antibiotic resistant was against carbapenem antibiotics and aminoglycosides specially amikacin. Similar results were reported by other researchers [10, 31, 33, 34]. A previously published review from Iran reported a raising pattern of antibiotic resistance rate science 1999 to June 2021in *Enterobacter* clinical isolates [33]. Comparison the results reported in this review with our finding also confirmed increasing rate of antibiotic resistance concerning different group of antibiotics including; 3rd generation cephalosporins (5.4% to 27.3%), cefepime (7.5%), ciprofloxacin (24.5%), imipenem (3.4%) and aztreonam (21.3%), from June 2021 till now.

Data analyzing showed, meanwhile, some antibiotic agents such as cefepime, carbapenems and different aminoglycoside antibiotics are still effective against non-MDR *E. cloacae* complex. High frequency of resistance was detected against majority of antibiotic agents in MDR isolates. Minimum antibiotic resistance in MDR isolates was detected against imipenem, meropenem and also amikacin (Table 4). In the recent decade, carbapenems like imipenem was reported as the most effective antibiotic agent against MDR *E. cloacae* complex and amikacin also mentioned as the most effective aminoglycoside against them [5, 6, 34, 35]. Thus, carbapenem antibiotics and amikacin could introduced as the most effective antibiotics against MDR *E. cloacae* complex. Even though, carbapenem resistance in *E. cloacae* complex also were reported repeatedly in the recent years [32].

The *E. hormaechei* was most prevalent species among studied *Enterobacter* species in the present study. Some other reports also have supported this finding [9, 36–38]. *E. cloacae* was the most frequent isolate after *E. hormaechei* in the present study. Some reports also introduced *E. hormaechei* and *E. cloacae* as the most common *Enterobacter* isolates from human clinical samples [6]. Statistical analysis also revealed that *E. hormaechei* isolates were significantly more susceptible to ceftriaxone and aztreonam in comparison to *E. cloacae.* It could be interpreted as higher ESBLs production or AmpC over production among *E. cloacae* isolates in comparison to the *E. hormaechei* isolates.

Antibiotic susceptibility findings were not significant different among various studied species. Therefore, similar susceptibility pattern in studied *Enterobacter* species could be concluded and similar antibiotic therapy regiment will be probably effective against infections caused by different species. However, supporting results have reported recently [6]. But our study population was very limited and only few reports existed in this respect. Thus, it is expected that it had to further investigated in the future to reach a clear conclusion.

The *rpos* gene had maximum prevalence among investigated virulence genes in the present study and similar results was reported also by Ghanavati et al., recently [8]. Data analysis showed that, blood cultivated isolates (11 isolates, 24.4%) were more virulent in comparison to other sample isolates and all virulence genes except *fepA* were more prevalent in blood isolates(data not showed). These isolates were categorized as *E. hormaechei* (6 isolates, 54.5%) and *E. cloacae* (5 isolates, 45.5%). The percentage increase calculators including; 23.8% for *csgA*, 106.8% for *csgD*, 17.2% for *fliI*, 23.6% for *fim*H, 3% for *rpoS* and 3.1% for *iutA* were detected for these isolates. Three of these 11 isolates were armed by all virulence genes and they were categorized as highly virulent isolates (two *E. hormaechei* and one *E. cloacae*). Regarding to these results, it could be concluded that *E. hormaechei* and *E. cloacae* are more virulent in comparison to *E. kobei* and *E. roggenkampii.* Ganbold et al., also reported same conclusion recently [9].

They were non-MDR and also were susceptible to majority of antibiotics except of beta-lactam agents (antibiotics with intrinsic resistance). This could be reasonable because, such blood colonizing isolates that are targeting directly by immune cell activities continuously, preferred to acquire many virulence properties that enable them to survive challenging conditions of surrounding harsh environment. Instead, they may acquire other genes such as antibiotic resistance genes with comparatively lower frequency or even tolerate some gene reductions specially regarding ARGs. Such fitness-cost phenomenon also was reported in *Pseudomonas aeruginosa* recently [39].

Based on the results, the *iutA* and *fepA* genes were significantly more frequent in *E. hormaechei* in comparison to other specie (Table 5)*.* The *iutA* and *fepA* genes categorized as enterobactin and aerobactin receptor genes respectively and they are superior to other siderophore receptors such as yersiniabactin or salmochelin receptors specially in an iron depleted environment [40]. Therefore, it could be estimated that *E. hormaechei* evolved to acquire iron more efficiently specially in a depleted iron environment and subsequently outcompete other species. We thought, it may explain higher prevalence of *E. hormaechei* in the human clinical samples in comparison to other species. Key role of aerobactin in virulence, biofilm and stress resistance of other Enterobacteriaceae member *Yersinia pseudotuberculosis* was also suggested recently [41]. Using iron acquisition systems as a carrier for antibiotic agents was hypothesized recently [42]. Daoud et al., reported that siderophore receptor *fepA* involved in delivering some drugs such as catechol-cephalosporins in to the bacterial cell recently [43]. Therefore, *fepA* gene could be a suitable target for designing novel anti-virulence/antibiotic agents against *E. hormaechei* infections*.*

The positive predictory role of *fepA* and negative predictory role of *csgD* for *E. hormaechei* against other studied species also was confirmed by logistic regression analysis. Therefore, condition expressing presence of *fepA* gene and absence of *csgD* gene could be regarded as a diagnostic key point for differentiation of *E. hormaechei* from other species.

Data analysis showed that *csgA*, *csgD* and *iutA* genes were associated with cefepime sensitivity (Fig. 2). The *csgD* gene was detected only in cefepime sensitive isolates. The *csgA* gene also was detected in very limited number (1 from 23, 4.3%) of cefepime resistant isolates. Both *csgA* and *csgD* genes involved production and control of curli fimbria and also, they promoting bacterial cells to shift from planktonic condition to biofilm former [13, 44]. Therefore, cefepime could introduced as a suitable antibiotic agent against biofilm associated infections caused by *Enterobacter* spp. However, it has to further investigated in bigger bacterial groups.

## Conclusion

Despite continuous increasing of antibiotic resistance in *Enterobacter cloacae* complex, the present study finding showed that carbapenem antibiotics and amikacin could be effective even against MDR isolates. Blood isolates were significantly more virulent but less resistant against many antibiotic agents in comparison to the other sample isolates. Our findings hypothesized that hyper evolution of some virulence properties such as iron acquisition systems in *E. hormaechei* could explain higher prevalence of this species in human clinical samples in comparison to the other species. Some virulence genes such as *csgA*, *csgD* were associated with cefepime susceptibility in ECC specially biofilm former isolates. For the first time, differential diagnostic role of some virulence genes such as *fepA* and *csgD* proposed and the *fepA* gene introduced as a suitable target for designing novel anti-virulence/antibiotic agents against *E. hormaechei* isolates. Collectively, we believe that in spite to many investigations some interaction between different genes may remained unknown. In addition to the environmental stresses, some genes may affect the expression of others and also acquisition rate of other genes directly or indirectly. But these interactions have to revealed during future studies and our study proposed some preliminary idea about these ambiguous interactions that may lead other investigations in a even direction that finally results to novel and clear conclusions and introducing suitable target for designing novel anti-virulence agents or new therapeutic regiments including combination of anti-virulence agents and antibiotics.

## Data Availability

The datasets used and /or analyzed during the current study are available from the corresponding author on reasonable request.

## Abbreviations

ECC: *Enterobacter cloacae* Complex
PCR: Polymerase Chain Reaction
MDR: Multidrug-Resistant
XDR: Extremely Drug Resistant
ESBL: Extended Spectrum Beta-Lactamase
CLSI: Clinical & Laboratory Standards Institute
SPSS: Statistical Package for the Social Sciences
VGs: Virulence Genes
ARGs: Antibiotic Resistance Genes
MOLDI-TOF: Matrix-assisted laser desorption/ionization -time-of-flame
URT: Upper Respiratory Tract
WHO: World Health Organization
