# Correction: Some virulence genes are associated with antibiotic susceptibility in *Enterobacter cloacae* complex

**DOI:** 10.1186/s12879-024-10060-5

**Published:** 2024-10-16

**Authors:** Fatemeh Mosaffa, Fereshteh Safari, Mahin Veisi, Omid Tadjrobehkar

**Affiliations:** Departement of Medical Microbiology (Bacteriology & Virology), Afzalipour School of Medicine, Kerman University of Medical Sciences, Kerman, Iran

**Correction to: BMC Infectious Diseases (2024) 24:711**

10.1186/s12879-024-09608-2

Following publication of the original article [1] the authors would like to correct a line in the introduction. The sentence originally read:

The *iutA* is encoding gene of Enterobactin receptor that delivered by type 3 secretion system (T3SS) into the host cells.

The sentence should read:

The *iutA* is encoding gene of ferric aerobactin receptor [19].

The original article has been corrected.
